# Three Types of Broadly Reacting Antibodies against Influenza B Viruses Induced by Vaccination with Seasonal Influenza Viruses

**DOI:** 10.1155/2018/7251793

**Published:** 2018-05-08

**Authors:** Daisuke Hirano, Nobuko Ohshima, Ritsuko Kubota-Koketsu, Ayami Yamasaki, Gene Kurosawa, Yoshinobu Okuno, Shunji Yoshida, Yoshikazu Kurosawa

**Affiliations:** ^1^Section of Rheumatology and Infectious Diseases, Department of Internal Medicine, Fujita Health University, Toyoake, Aichi 470-1192, Japan; ^2^Center for Collaboration in Research and Education, Fujita Health University, Toyoake, Aichi 470-1192, Japan; ^3^Department of Innovation for Advanced Medicine, Center for Research Promotion and Support, Fujita Health University, Toyoake, Aichi 470-1192, Japan; ^4^Clinical Research Section, Research Department, The Research Foundation for Microbial Disease of Osaka University, Suita, Osaka 565-0871, Japan; ^5^Division of Innovation for Advanced Medicine, Institute for Comprehensive Medical Science, Fujita Health University, Toyoake, Aichi 470-1192, Japan; ^6^Department of Academic Research Support Promotion Facility, Center for Research Promotion and Support, Fujita Health University, Toyoake, Aichi 470-1192, Japan; ^7^Osaka Institute of Public Health, Osaka, Osaka 537-0025, Japan

## Abstract

We analyzed the antibody (Ab) repertoire against influenza B viruses induced by vaccination with seasonal influenza viruses in one individual who had never been vaccinated until 2009. The vaccine used in this study comprised B/Massachusetts/2/2012 (Yamagata lineage), A/Texas/50/2012 (H3N2), and A/California/7/2009 (H1N1). One month after the subject received two vaccinations, blood (200 ml) was obtained and peripheral mononuclear cells were prepared, and a large Ab library was constructed using phage display technology. The library was screened with HA-enriched fraction of B/Massachusetts/2/2012 and B/Brisbane/60/2008 (Victoria lineage) virus, and a total of 26 Abs that potentially bound to hemagglutinin (HA) molecules were isolated. Their binding activities to six influenza B viruses, three of Yamagata lineage and three of Victoria lineage, and two influenza A viruses, H1N1 and H3N2, were examined. The Abs showed cross-reactivity at three different levels. The first type bound to all Yamagata lineage viruses. The second type bound to both Yamagata and Victoria lineage viruses. The third type bound to both influenza A and B viruses. These results indicate that common epitopes exist on HA molecules of influenza virus at various levels, and humans have capability to produce Abs that bind to such common epitopes.

## 1. Introduction

Influenza is an infectious disease of the respiratory tract that affects millions of people annually [1]. Since antibodies (Abs) play important roles in protection against influenza virus, preventive vaccines have been considered one of the most efficient measures to control viral infection [2]. Hemagglutinin (HA) is the main target of influenza virus-neutralizing Abs. However, viruses can acquire mutations in the epitopes recognized by such Abs, leading to viral escape and a potential epidemic in the following year. Therefore, vaccine strains need to be changed almost annually to remain effective. As long as the vaccine strain is a good match for the circulating virus, vaccination is effective for preventing viral infection [3]. However, the mode of response against influenza virus infection seems to be heterogeneous among humans [4]. Some individuals do not contact influenza without vaccination for a long time, whereas others often contact influenza even with annual vaccination. The reason for this difference in susceptibility to infection remains unclear, but one possibility is differences in the ability of Abs produced by memory B cells [5, 6]. It has been well established that natural influenza virus infection can provide cross-reactive immunity that can reduce the impact of infection with distinct influenza A strains and subtypes, including H1N1, H3N2, H2N2, H5N1, and H7N9 [7]. In the present study, we analyzed the repertoire of Abs against influenza viruses in a person who did not contact influenza without vaccination for a long time.

To analyze such Ab repertoire, we developed the following experimental strategy [8]. First, a large number of B lymphocytes are collected by apheresis from a donor, and an Ab library is constructed using phage display technology. Second, the library is screened with HA-enriched fraction of virus particles, and clones with binding activity to HA are isolated. Finally, neutralizing activity and strain specificity of the respective clones are analyzed. In previous studies, we collected large number of B cells by apheresis [5, 8, 9]. The pooled fraction contained approximately 10^9^ B lymphocytes. We then constructed an Ab library composed of approximately 3 × 10^10^ independent clones. In the present study, however, vaccination was performed twice before blood collection. Since we expected the presence of a relatively large number of B cells in the peripheral blood whose growth had been induced or stimulated by vaccination with specific viruses [10], we collected 200 ml peripheral blood and prepared mononuclear cells instead of 3 l blood-equivalent B cells by apheresis. After we constructed an Ab library that could cover the Ab repertoire present in the collected B cells, we screened the library with HA-enriched fraction of virus particles from three strains, B/Massachusetts/2/2012, B/Brisbane/60/2008, and A/Uruguay/716/2007 (H3N2). The results of clones isolated by screening with influenza type B viruses are reported.

## 2. Materials and Methods

### 2.1. Seasonal Influenza Vaccine

Seasonal influenza vaccine from 2013 to 2014 in Japan which was composed of three strains, B/Massachusetts/2/2012, A/Texas/50/2012 (H3N2), and A/California/7/2009 (H1N1) was used for vaccination. This vaccine (lot number: HE39A) was produced by the Research Foundation for Microbial Disease, Osaka University.

### 2.2. Viruses

The following influenza virus strains were used: B/Yamagata lineage: B/Massachusetts/2/2012 (MAS), B/Wisconsin/1/2010 (WIS), and B/Phuket/3073/2013 (HKT); B/Victoria lineage: B/Brisbane/60/2008 (BRI), B/Malaysia/2506/2004 (MYS), and B/Texas/2/2013 (TEX). A/H1N1: A/California/7/2009. A/H3N2: A/Texas/50/2012. These virus strains were used for vaccine production in Japan, and the undiluted solutions of the vaccines were provided from Kannonji Institute, Research Foundation for Microbial Diseases of Osaka University. They were prepared in embryonated hen eggs by inoculating the vaccine strains, and the virus particles were purified by means of sucrose gradient centrifugation. The purified virus particles were treated with ether to remove lipid, and formalin was added. Ether- and formalin-treated samples were subjected to sucrose gradient centrifugation again. The HA-enriched region was collected for preparation of split influenza vaccines. These HA molecules were used for screening and characterization in this project.

### 2.3. Monoclonal Abs (mAbs)

Mouse mAbs 3A12 which reacts with B/Yamagata lineage strains [11] and 10B8 which reacts with B/Victoria lineage strains [12] stocked in the Okuno's laboratory were used. The following three human mAbs were used for comparison as Fab-PP (P denotes a single Fc-binding domain of protein A) form of Abs: anti-H3 Ab, F045-092 [9]; anti-H1 Ab, F081-007 [5]; and anti-VP4 of rotavirus, 2-3E [13].

### 2.4. Construction of the Ab Library

A large combinatorial Ab library was constructed using the phage display method as described previously [14]. Briefly, 1.68 × 10^8^ B lymphocytes in 200 ml peripheral blood cells were collected from the donor, and heavy (H) and light (L) chain libraries were constructed. Finally, H and L chains were combinatorially assembled. The resulting Ab library contained 2.18 × 10^9^ independent clones.

### 2.5. Screening of the Ab Library

Phages bound to HA-enriched fraction of virus particles were selected by a panning method as described previously [8]. In brief, HA-enriched fraction of virus particles of B/Massachusetts/2/2012 and B/Brisbane/60/2008 strains was used as antigens (Ags) in the screenings. After panning three times, eluted phages were used to infect *E. coli* (DH12S) cells. Infected *E. coli* cells were plated onto Luria-Bertani agar plates containing 100 *μ*g ampicillin/ml and 0.2% glucose. *E. coli* colonies harboring phagemid were picked and grown in 2x YT medium containing 100 *μ*g/ml ampicillin, 0.05% glucose, and 1 mM isopropyl-*β*-D-thiogalactopyranoside at 30°C overnight. During growth of *E. coli*, the Fab-cp3 (cp3 denotes coat protein III on filamentous phage M13) form of Ab was secreted into the medium [15]. Culture supernatants containing Fab-cp3 molecules were subjected to enzyme-linked immunosorbent assay (ELISA) against influenza B viruses used as Ags in the screening process.

### 2.6. ELISA

HA-enriched fraction of virus particle was coated onto 96-well MaxiSorp immunoplates (Nunc). After blocking with 5% bovine serum albumin (BSA), Fab-cp3 Ab in the supernatant of *E. coli* cultures was added to each well. The plates were then incubated with mouse anti-cp3 Ab, followed by subsequent incubation with peroxidase-conjugated goat anti-mouse IgG (H + L chain, MBL). Horse radish peroxidase (HRP) substrate (1-Step Ultra TMB- (3,3′,5,5′-tetramethylbenzidine-) ELISA; Thermo Fisher Scientific) was then added to each well, and color development was monitored. The peroxidase reaction was stopped by the addition of 2 N H_2_SO_4_, and absorbance at 450 nm was measured.

### 2.7. Preparation of mAbs

Phagemid DNAs from isolated Ab clones were digested with SalI and then self-ligated to convert the Fab-cp3 form into the Fab-PP form (P denotes a single Fc-binding domain of protein A) [16]. Fab-PP Abs were purified with IgG Sepharose (GE Healthcare).

### 2.8. Titration of Binding Activity

HA-enriched fraction of virus particles was coated onto 96-well MaxiSorp immunoplates (Nunc). After blocking with 5% BSA, purified Fab-PP Ab was added at various concentrations between 0.005 and 20 *μ*g/ml to each well and then incubated. After washing, goat anti-human IgG (Fab-specific) peroxidase (Sigma-Aldrich) was added and incubated. After washing, HRP substrate (1-Step Ultra TMB-ELISA; Thermo Fisher Scientific) was added to each well, and color development was monitored. The peroxidase reaction was stopped by the addition of 2 N H_2_SO_4_, and absorbance at 450 nm was measured.

### 2.9. SDS-PAGE and Western Blotting

HA-enriched fraction of virus particles was separated by SDS-PAGE under nonreducing conditions. Protein bands were stained with Coomassie brilliant blue (CBB). They were also transferred to an Immobilon-P membrane (Millipore) for Western blotting. The membrane was incubated with Fab-cp3 Abs. For detection, mouse anti-cp3 Ab (MBL) was used as the primary Ab, and peroxidase-conjugated goat anti-mouse IgG (H + L chain; MBL) was used as the secondary Ab.

### 2.10. Hemagglutination Inhibition (HI) Assay

The HI assay was performed as described previously [9]. In brief, serial dilutions of 200 *μ*g/ml of purified Fab-PP in PBS were prepared. Serial dilutions of Fab-PP were preincubated with 4 HA units of virus per well. Guinea pig red blood cells (0.75%) in PBS were added to each well, and the plate was incubated at room temperature for 60 min. The results shown indicate the lowest concentration (*μ*g/ml) of Fab-PP Ab to inhibit hemagglutination.

### 2.11. Immunostaining of HA1-Expressing Cells

#### 2.11.1. Construction of HA Expression Vector

The polypeptide encoding HA1 region of B/Massachusetts/2/2012 virus was expressed on 293T cells as follows. The DNA segment encoding HA1 region was prepared by PCR using artificially constructed HA gene B/Massachusetts/2/2012 as template DNA and two primers SfiI FluBMas12HA F primer 5′-TTAGGCCCAGCCGGCCGACAGAATATG and FluBMas12HA345 XbaR primer 5′-GTCTCTAGACTCCTTCAGCAGTTTAGC. Then, HA1 with Flag tag was inserted into pDisplay vector (Thermo Fisher Scientific), resulting in BMas12HA1/pDisplay.

#### 2.11.2. Immunostaining

A round circle cover glass was put into each well of a 24-well plate, and 0.1% gelatin in PBS was added. After incubation for 30 min at 37°C, each well was washed with PBS. 293T cells were added and incubated at 37°C overnight in the presence of 5% CO_2_. BMas12HA1/pDisplay was transfected into 293T cells using Lipofectamine 3000 (Thermo Fisher Scientific), and the cells were incubated at 37°C overnight. After washing with PBS, cells were fixed with 4% PFA (paraformaldehyde). After washing with PBS, cells were blocked with 2.5% BSA in PBS. As the first Ab, Fab-PP Ab, mouse anti-myc tag Ab (MBL) and mouse Ab 3A12 were added and incubated. After washing with PBS, Alexa Fluor 488 anti-human IgG (Thermo Fisher Scientific) for Fab-PP Ab or Alexa Fluor 488 anti-mouse IgG (Thermo Fisher Scientific) for anti-myc tag and 3A12 was used as the second Ab. After washing with PBS, the cells on the glass were taken out and observed by fluorescent microscopy (biological microscope, Olympus BX50).

### 2.12. Competitive ELISA with Mouse mAbs

To identify the epitopes recognized by Abs showing HI activity, competitive ELISA between mouse mAbs whose epitopes are known and the Fab-cp3 form of Abs isolated in this study was performed as follows. HA-enriched fraction of virus particles was coated onto a 96-well MaxiSorp immunoplate. Fab-cp3 molecules in the supernatant of *E. coli* cultures were concentrated 20-fold and used as a competitor, and mouse mAbs 3A12 and 10B8 were used for detection of binding to virus particles. A total of 50 *μ*l of 3A12 or 10B8 at an optimized concentration were mixed with 50 *μ*l of 20-fold concentrated Fab-cp3, and the mixture was added to a HA-coated well and incubated. Each well was incubated with peroxidase-conjugated goat anti-mouse IgG (H + L chain; MBL) as a secondary Ab, and HRP substrate (1-Step Ultra TMB-ELISA; Thermo Fisher Scientific) was added to each well. The peroxidase reaction was stopped by the addition of 2 N H_2_SO_4_, and absorbance at 450 nm was measured.

### 2.13. Competitive ELISA among Isolated Clones

Competitive ELISA was performed as described previously [5]. The Fab-PP form of Abs was used for detection of binding activity to HA-enriched fraction of virus particles, and the Fab-cp3 form of Ab was used as a competitor. Fab-cp3 molecules in the supernatant of *E. coli* cultures were concentrated 20-fold before use. HA-enriched fraction of virus particles was coated onto a 96-well MaxiSorp immunoplate. A total of 50 *μ*l of Fab-PP at an optimized concentration were mixed with 50 *μ*l of 20-fold concentrated Fab-cp3, and the mixture was added to a virus-coated well. Peroxidase-conjugated rabbit anti-sheep IgG (ROCKLAND) was then added to each well as a secondary Ab. After washing, HRP substrate (1-Step Ultra TMB-ELISA; Thermo Fisher Scientific) was added, and color development was monitored. The peroxidase reaction was stopped by the addition of 2 N H_2_SO_4_, and absorbance at 450 nm was measured.

### 2.14. Immunoprecipitation Followed by Western Blotting

The Fab-PP form of Ab was mixed with HA-enriched fraction of virus particles and incubated at room temperature for 1 h. IgG Sepharose 6 Fast Flow (GE Healthcare) was added, and the mixture was rotated for 1.5 h at 4°C. After incubation, IgG Sepharose was washed, and SDS-PAGE sample buffer was added. The mixture was heated at 95°C for 3 min. The proteins were separated by SDS-PAGE and transferred to PVDF (polyvinylidene difluoride) membrane. After blocking, the membrane was reacted with 3A12 antibody, followed by reaction with anti-mouse IgG-HRP (MBL). The substrate for HRP, Luminata Forte (Millipore), was added, and the immunological band was detected by CCD camera.

### 2.15. Neutralization Assay

For measurement of virus-neutralizing activity, a focus reduction assay was performed [17]. Fab-PP Abs (250 or 100 *μ*g/ml) were mixed with an equal volume of 100 focus-forming units (FFU) of influenza virus and applied to MDCK (Madin-Darby canine kidney) cells in a 96-well plate. After incubation with the mixture, the cells were washed with serum-free MEM and cultured in MEM containing 0.4% BSA at 37°C for 15 h. The cells were then fixed with ethanol and stained with a peroxidase and anti-peroxidase complex. The number of foci containing one or more cells was counted. The results are presented as the focus reduction rate (%).

### 2.16. Nucleotide Sequence Accession Number

**Figure 1:**
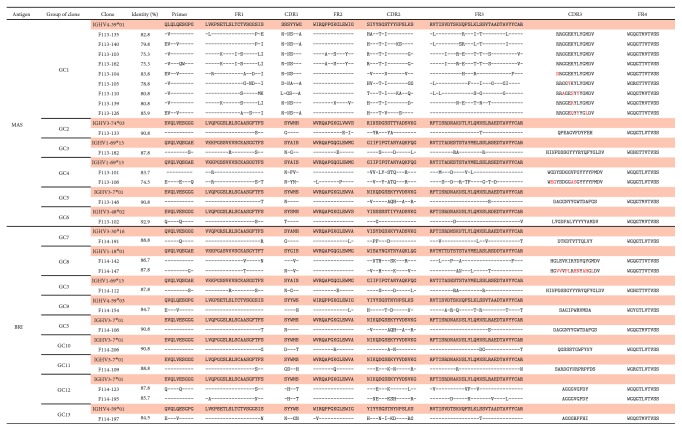
Amino acid sequences of the V_H_ region of the 26 Abs isolated in this study. Germline V_H_ genes in the respective clones were assigned and their amino acid sequences are shown. Dash indicates the same amino acid as that of the germline gene. Amino acids that differ from that of the germline gene are given. Identity (%) indicates the degree of similarity between the germline gene and that of the respective Ab.

**Figure 2:**
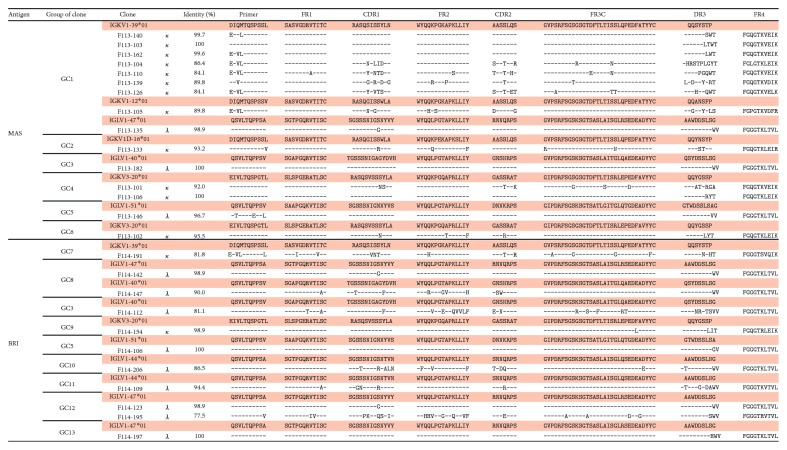
Amino acid sequences of the V_L_ region of the 26 Abs isolated in this study. Germline V_L_ genes, *κ* or *λ*, in the respective clones were assigned and their amino acid sequences are shown. Dash indicates the same amino acid as that of the germline gene. Amino acids that differ from that of germline gene are given. Identity (%) indicates the degree of similarity between the germline gene and that of the respective Ab.

Nucleotide sequences of the variable regions of H and L chains of Abs isolated in this study have been deposited in the DDBJ database (accession numbers LC279543–LC279568 for the H chains in Figure 1 and LC279569–LC279594 for the L chains in Figure 2).

## 3. Results

### 3.1. Isolation of 26 Abs Binding to Influenza B Virus

We isolated Abs that potentially bound to HA of influenza B viruses as follows. The blood donor in the present study was the same donor described in our previous study [5]. He was born in 1947 and contacted influenza several times during childhood (possibly H1N1 and H2N2) and in 1968 (probably H3N2). For 46 years afterward, he was never in bed with influenza, and he had never been vaccinated until 2009. In the previous study, the donor was vaccinated with A/California/7/2009 (H1N1) in November 2009, and many Abs that bound to HA of HIN1 viruses were isolated through construction of a large Ab library [5]. In the present study, vaccination was performed twice at 2-week intervals in April 2014, and blood was collected 1 month later. The vaccine used in this study comprised three virus strains: B/Massachusetts/2/2012, A/Texas/50/2012 (A/H3N2), and A/California/7/2009 (A/H1N1). An Ab library was constructed using phage display technology from 1.68 × 10^8^ B lymphocytes in 200 ml peripheral blood cells. The size of the V_H_ library was 1.08 × 10^9^ clones, the size of the V_L_ library was 3.59 × 10^7^ clones, and the size of the Ab library was 2.18 × 10^9^ clones. The Ab library was screened by panning with HA-enriched fraction of B/Massachusetts/2/2012 (Yamagata lineage) virus particles and separately with B/Brisbane/60/2008 (Victoria lineage) virus particles. After the third round of panning with the respective viruses, 144 phage clones were picked up from each screening. Binding activity of respective clones to HA-enriched fraction of the virus particles that had been used for screening was further examined. Finally, 81 clones isolated by screening with B/Massachusetts/2/2012 and 29 clones isolated by screening with B/Brisbane/60/2008 were judged to be candidates for anti-HA Abs against influenza B viruses.

The sequences of the V_H_ gene of respective clones were determined and classified as follows. The 81 clones isolated by screening with B/Massachusetts/2/2012 comprised 15 different clones, and the 29 clones isolated by screening with B/Brisbane/60/2008 comprised 11 different clones (Table 1). The sequence of complementarity-determining region 3 (CDR3) of the H chain was utilized for further classification. If the length is the same and the sequence is identical or very similar between clones, it is likely that the clones were derived from the same original B cell and diverged by introduction of mutations. Based on this interpretation, the clones were further classified into group of clone (GC). Since F113-182 and F114-112, as well as F113-146 and F114-106, were derived from the same original B cells, the 26 Abs were classified into 13 GCs.

Figure 1 shows the amino acid sequences of V_H_ fragments in 15 different Abs isolated by screening with B/Massachusetts/2/2012 and those of 11 different Abs isolated by screening with B/Brisbane/60/2008. Germline V_H_ genes were assigned according to sequence identity. Since the degree of identity ranged from 74 to 91% except for one clone, F113-102 (92.9%), we speculated that not all isolated clones, including the above clone, were derived from B cells that had been newly produced by vaccination, but instead derived from memory B cells that had been present before vaccination. Figure 2 shows the amino acid sequences of V_L_ fragments in all 26 Abs. We classified clones using V_H_ sequences based on our following experience. Clones whose V_H_ sequence and binding specificity were identical to each other demonstrated that V_L_ sequences were also identical or very similar to each another. This experience was also applicable to almost all 26 Abs (Figures 1 and 2).

### 3.2. Classification of Abs into Three Types Based on Strain Specificity of the Binding Activity

**Figure 3:**
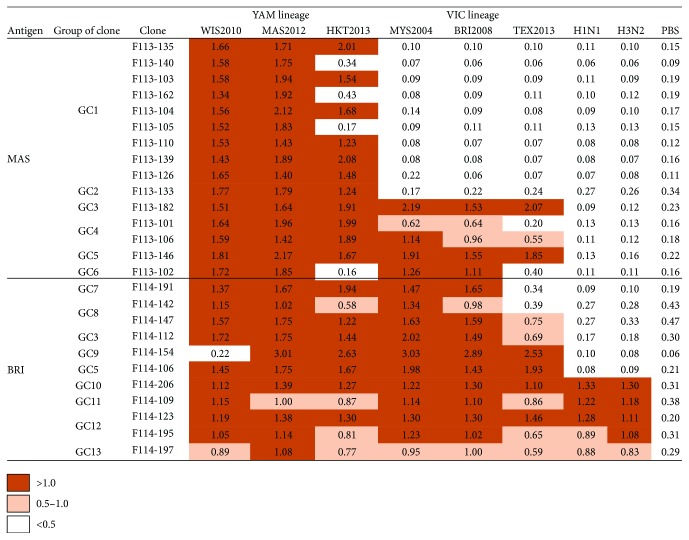
Binding activities measured by ELISA between eight virus particles and the 26 Abs isolated in this study. Viruses tested were WIS2010, B/Wisconsin/1/2010; MAS2012, B/Massachusetts/2/2012; HKT2013, B/Phuket/3073/2013; MYS2004, B/Malaysia/2506/2004; BRI2008, B/Brisbane/60/2008; TEX2013, B/Texas/2/2013; H1N1, A/California/7/2009; and H3N2, A/Texas/50/2012. Absorbance values > 1 are marked by light brown, and values 0.5–1 are marked by faint light brown.

We next examined the binding activity of the 26 Abs against eight different influenza viruses: B/Yamagata lineage: B/Wisconsin/1/2010, B/Massachusetts/2/2012, and B/Phuket/3073/2013; B/Victoria lineage: B/Malaysia/2506/2004, B/Brisbane/60/2008, and B/Texas/2/2013; H1N1 virus: A/California/7/2009; and H3N2 virus: A/Texas/50/2012 by ELISA. None of the clones bound only to B/Massachusetts/2/2012, which had been included in the vaccine (Figure 3). This seemed to be consistent with the finding that there was no clone that was derived from B cells newly induced by vaccination. The isolated clones were classified into three types. Type 1 comprised nine clones belonging to GC1 and one clone belonging to GC2 that bound to the three B/Yamagata lineage viruses. Among them, F113-140, F113-162, and F113-105 bound weakly to B/Phuket/3073/2013. Type 2 comprised eleven clones belonging to GC3–9 bound to both B/Yamagata and Victoria lineage viruses. However, some clones such as F113-102, F114-191, and F114-142 bound only weakly to B/Texas/2/2013. Type 3 comprised five clones of GC10–13 bound to both influenza B viruses and H1N1 and H3N2 viruses, although the binding activity of F114-197 was weak. Interestingly, these four clones used the same germline V_H_ gene, V_H_3-7∗01 (Figure 1). Furthermore, while they utilized different germline V_L_ genes, V*_λ_*1-44∗01 or V*_λ_*1-47∗01, these two germline genes only showed differences at a few residues (Figure 2).

**Figure 4:**
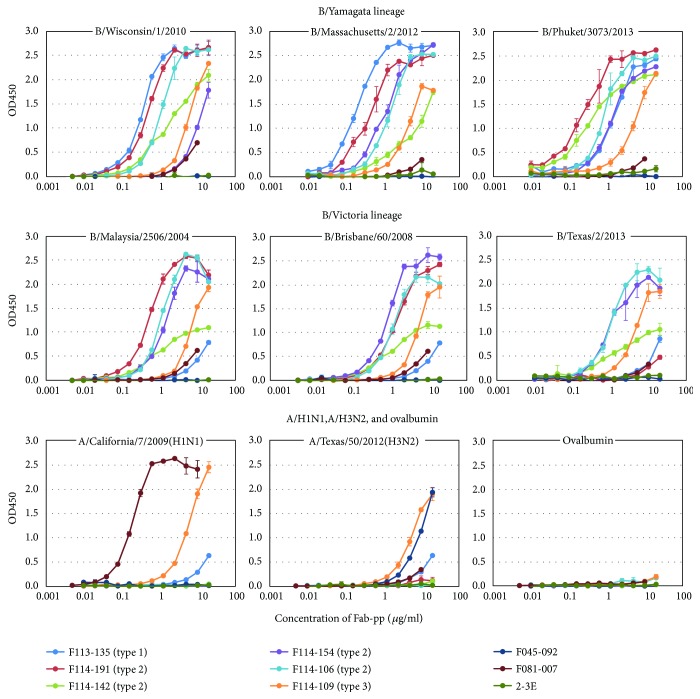
Titration of binding activity of representative Abs against various kinds of influenza viruses. Purified Fab-PP form of Abs, F113-135 (type 1), F114-191, F114-142, F114-154, F114-106 (type 2), F114-109 (type 3), F045-092 (anti-H3 Ab), F081-007 (anti-H1 Ab), and 2-3E (anti-VP4 of rotavirus antibody) were utilized in this experiment. The binding activities to six kinds of influenza B virus, one H1N1 virus, one H3N2 virus, and ovalbumin (negative control) were examined under various concentrations.

In order to analyze the binding activity quantitatively, Fab-PP form of Abs was prepared. Since P corresponds to a single Fc-binding domain of protein A, they could be easily purified. The binding activity of Fab-PP Abs to HA-enriched form of eight different virus particles was examined. Since 1 *μ*g/ml Fab approximately corresponds to 0.02 *μ*M, the binding activity of monovalent Ab was observed under the concentration between nM and *μ*M (Figure 4). F113-135 classified into type 1 strongly bound to three viruses belonging to Yamagata lineage. F114-191 classified into type 2 strongly bound to five viruses belonging to Yamagata and Victoria lineages except for B/Texas/2/2013. These results were consistent with the data shown in Figure 3. Other three clones F114-142, 114-154, and F114-106 belonging to type 2 bound to Yamagata and Victoria lineage viruses in similar specificity as shown in Figure 3. F114-109 belonging to type 3 bound to all the viruses including H1N1 and H3N2 although with relatively weak binding activity.

### 3.3. Abs Classified into Type 1 Show HI Activity and Bind to the 150 Loop of HA

**Figure 5:**
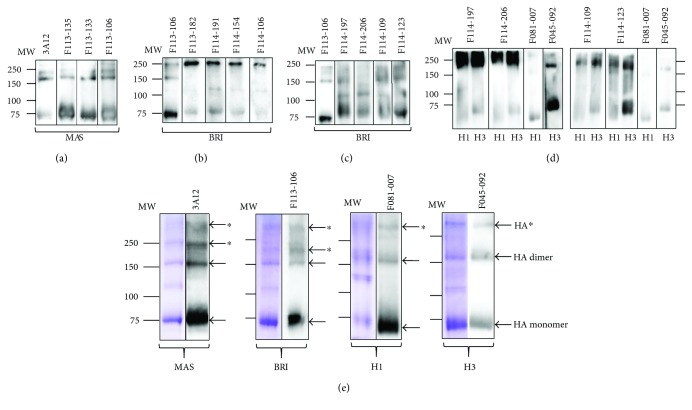
Western blotting analysis of representative Abs against influenza B and A virus strains. (a) Three Abs that showed HI activity. Mouse mAb 3A12 was used as a positive control against influenza B/MAS. (b) Four Abs belonging to type 2. (c, d) Four Abs belonging to type 3. (d) F045-092 and F081-007 were used as a positive control against influenza A/H3N2 and influenza A/H1N1, respectively. (e) Left lane, HA-enriched fraction of virus particles was applied and stained with CBB. Right lane, Western blotting with four mAbs. HA^∗^, the position of HA monomer and dimer were clear. However, while HA should be included in the upper bands, it is possible that something other than HA molecule is included.

**Figure 6:**
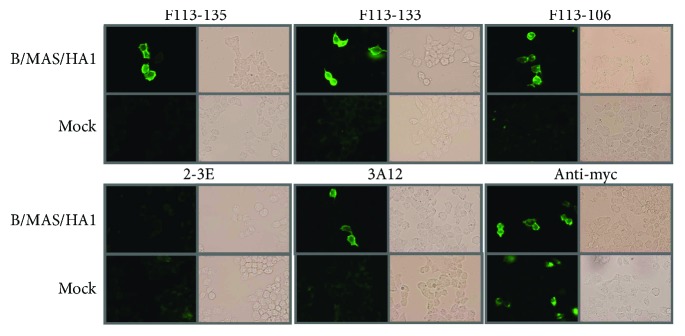
Immunostaining of cells that express HA1 molecule on the cell surface. Fab-PP form of Abs, F113-135 (type 1), F113-133 (type 1), and F113-106 (type 2) bound to HA1 expressed on the cell surface. Positive control: 3A12, anti-B/HA Ab, 2-3E (Fab-PP form), anti-rota VP4 Ab, anti-myc Ab for system check. Mock, pDisplay vector without insert was used for transfection.

HI activity was examined for 22 Abs belonging to 13 GCs. Seven GC1 Abs, one GC2 Ab, and two GC4 Abs showed HI activity (Table 2). These Abs demonstrated positive signals at the position of HA by Western blotting analysis (Figure 5(a)). One GC6 Ab, F113-102, also showed HI activity albeit extremely low. The following experiment directly shows that the epitope recognized by Abs that possess HI activity is located on HA1. HA1 of B/Massachusetts/2/2012 was artificially expressed on the cell surface and F113-135 and F113-133 belonging to type 1 clearly bound to the surface of HA1-expressing cells. Even F113-106 belonging to type 2 gave a positive result (Figure 6).

**Figure 7:**
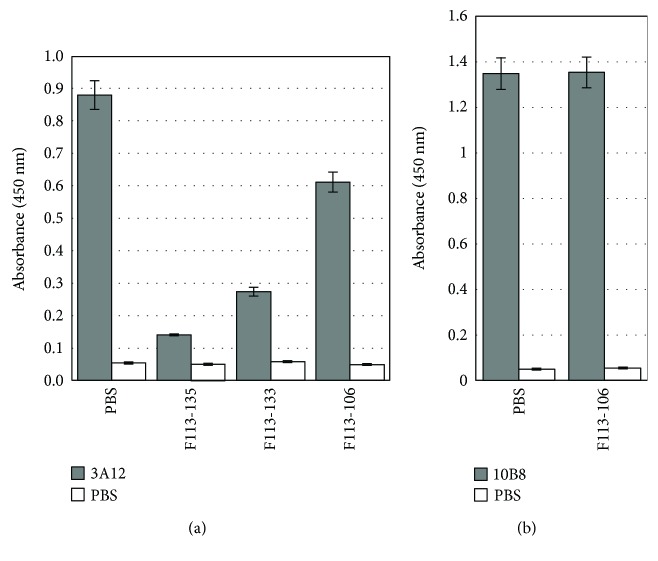
Competitive ELISA with mouse mAbs 3A12 and 10B8. (a) The binding activity of 3A12 to B/Massachusetts/2/2012 was examined in the presence of excess Fab-cp3 form of three anti-HA Abs, F113-135, F113-133, and F113-106. (b) The binding activity of 10B8 to B/Brisbane/60/2008 was examined in the presence of excess F113-106.

To identify the epitope recognized by these Abs, we performed competitive inhibition of binding with mAbs whose epitopes are known. MAb 3A12 reacts with B/Yamagata lineage strains, but not B/Victoria lineage strains [11]. The analysis of 3A12-escape mutants indicated that residues 141, 147, and 148 form a part of the epitope, and these residues are located at proximity to each other in the 150 loop of HA [11]. MAb 10B8 reacts with B/Victoria lineage strains, but not B/Yamagata lineage strains [12]. The analysis of 10B8-induced variants indicated that residues 164, 165, and 203 are located at proximity to each other and formed a part of the epitope [18]. They are located close to the receptor-binding site. Both F113-135 (GC1) and F113-133 (GC2) competed well with 3A12 for binding with B/Massachusetts/2/2012 virus particles (Figure 7). Thus, the epitope recognized by Abs classified as type 1 is located at the 150 loop of HA. F113-106 (GC4), which was classified as a type 2 Ab but showed HI activity, competed weakly with 3A12. This result suggests that the epitope recognized by F113-106 partly overlaps the epitope recognized by 3A12. However, F113-106 did not compete with 10B8 at all.

### 3.4. There Are at Least Four Common Epitopes on HAs of Both Yamagata and Victoria Lineage Viruses

While Abs that showed HI activity should bind to HA, the epitopes recognized by Abs that did not show HI activity could be present on something other than HA. Western blotting of type 2 Abs was performed using HA-enriched fraction of B/Brisbane/60/2008 virus particle. The positive signals were observed at HA position although the intensity of bands at monomer position was weak as shown in Figure 5(b). In order to examine the reason why positive signals detected by type 2 were located at high molecular weight position, we performed the following experiment. The distribution of all the proteins and that of HA molecules in the Ags which we had used was examined by gel electrophoresis followed by Western blotting. The results were shown in Figure 5(e). Left lane was the results of staining with Coomassie brilliant blue (CBB), and the right lane indicated the bands detected by four mAbs. 3A12 is a typical mouse anti-HA Ab used in this study. F081-007 is a human mAb that is a typical VH1-69 Ab detecting A type group 1 HAs. F045-092 is a human mAb that binds to H3 type HA. Major bands in the left lane corresponded to the positive bands detected anti-HA Abs. The faint band which seems to correspond to NA is also seen. Since the Ags used in the present study were treated with formalin, a part of HA molecules migrated to dimer and trimer positions. Furthermore, a part of HA migrated to even higher position. Major bands detected in Figure 5(b) seemed to correspond to HA trimers. The reason why the preference to monomer versus trimer exists could be derived from the stability of the epitope in the process of SDS-PAGE followed by Western blot. The epitope cannot be stably kept in monomer form but that the epitope is stabilized in multimer HA cross-linked by treatment with formalin.

**Figure 8:**
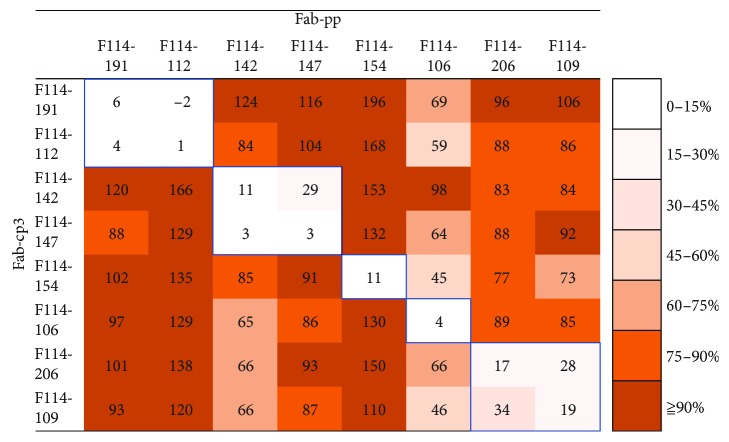
Competitive ELISA among isolated clones. The binding activity of Fab-PP Ab (indicated at the top) to B/Massachusetts/2/2012 virus particles was measured by ELISA in the presence of a 10-fold greater concentration of Fab-cp3 Ab (indicated at the left). F008-009, which is not an anti-HA Ab, was used as a control. The binding activity of Fab-PP in the presence of Fab-cp3 Ab was indicated as relative values as follows: the absorbance value in the presence of F008-009cp3 was set as 100% binding and the binding activity in the presence of various Fab-cp3 forms of Abs is indicated as % values.

To estimate the number of epitopes recognized by type 2 Abs, we performed competitive ELISA by combination of six different type 2 Abs without HI activity. The Fab-PP form of Ab was used for detection of binding activity to HA. The Fab-cp3 form of Ab was prepared as a competitor. Since Fab-cp3 form of Ab was secreted into the medium during growth of *E. coli*, they were concentrated 20-fold before use without purification. Fab-PP of Ab was used after purification. The binding activity of Fab-PP form of Ab to HA-enriched fraction of B/Massachusetts/2/2012 virus particles was measured by ELISA in the presence of much larger concentration of Fab-cp3 form of Ab. F114-191 (GC7) and F114-112 (GC3) share the epitope (Figure 8). Furthermore, F114-142 (GC8) and F114-147 (GC8) also share the epitope. However, F114-154 (GC9) and F114-106 (GC5) bind to independent epitopes. These results suggest that there are at least four common epitopes present on HAs of both Yamagata and Victoria lineage viruses.

### 3.5. Presence of a Common Epitope on HA of Both Influenza A and B Viruses

Isolation of type 3 Abs suggests the presence of common epitope(s) on both influenza A and B viruses. To examine whether the epitope is located on the HA molecule, the four kinds of type 3 Ab were subjected to Western blotting against B/Brisbane/60/20082 viruses (Figure 5(c)). While positive signals were observed in all four cases, the position is slightly higher than that of F113-106, which was type 2 Abs showing HI activity. Since these clones bound not only to influenza B viruses, but also to influenza A viruses, Western blotting of type 3 Abs was performed by using H1 and H3 virus particles. In this case, the major bands were located at high molecular weight position as the same as type 2 Abs. While the intensity was weak, the positive bands were also observed at the position of HA monomer. As described in the results of Western blot by type 2 Abs, we supposed that these results might be derived from the stability of the epitope.

**Figure 9:**
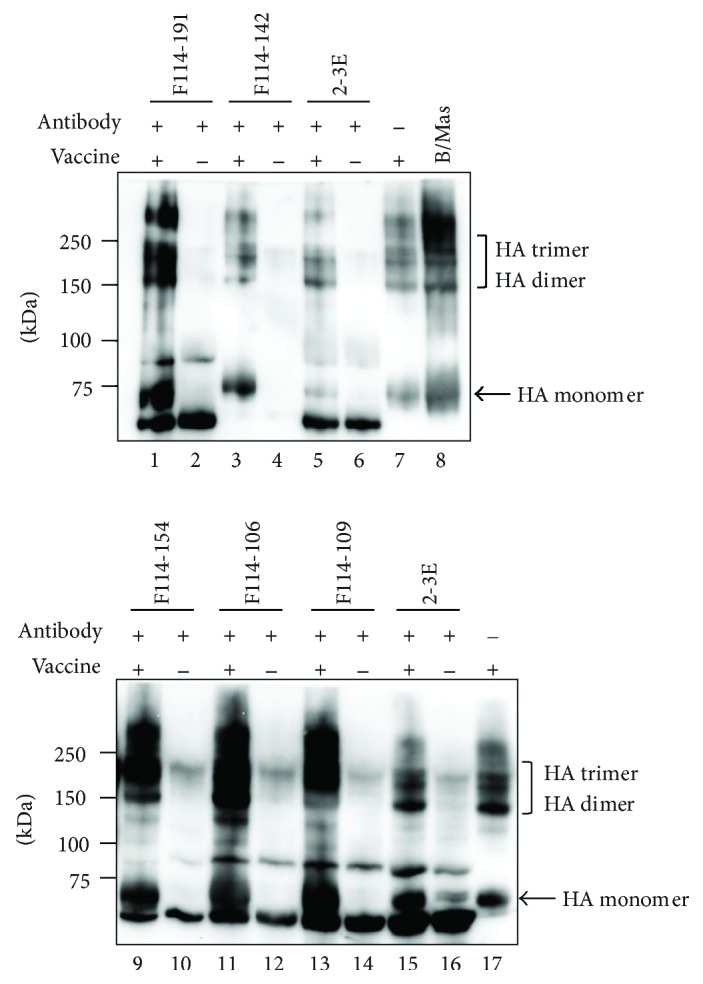
Western blotting of virus components complexed with Fab-PP form of Abs. Four type 2 Abs, F114-191, F114-142, F114-154, and F114-106, and one type 3 Ab F114-109 were analyzed. Vaccine was B/Massachusetts/2/2012. 2-3E, anti-VP4 of rotavirus Ab was a negative control.

To directly examine whether the epitope recognized by type 3 Abs is located on HA, we performed the experiment similar to that shown in Figure 6. However, type 3 Abs did not bind to HA artificially expressed on the cell surface (data not shown). Then, we newly designed an experiment as follows. The fact that multiple clones had been isolated according to our strategy indicated that type 3 Abs should bind to something in the HA-enriched fraction of virus particles used as Ag in the screening. Therefore, it should be possible to precipitate the substance by using Fab-PP form of Abs classified into type 2 and type 3. The precipitates were subject to gel electrophoresis followed by Western blotting with Ab that can detect HA. The results indicated that the major material precipitated by type 3 Abs is located at high molecular weight position (Figure 9). Similar results were obtained by using type 2 Abs. The results shown in Figure 5 and those in Figure 9 were consistent to each other. Although we could not think that this is a direct evidence, it will be possible that the epitopes recognized by Abs classified into type 2 and type 3 are located on HA.

To examine whether Abs classified into GC10–13 bind to the same epitope or to different epitopes, competitive ELISA was performed using four clones. As shown in Figure 8, F114-206 (GC 10) and F114-109 (GC 11) likely share the same epitope. This result was reasonable, since both clones utilized the same set of germline V genes, V_H_3-7∗01 for H chain and V*_λ_*1-44∗01 for L chain (Figures 1 and 2). On the other hand, F114-123 (GC 12) and F114-197 (GC 13) did not give clear results (data not shown).

### 3.6. Most Type 1 and Type 2 Abs Have Virus-Neutralizing Activity

**Figure 10:**
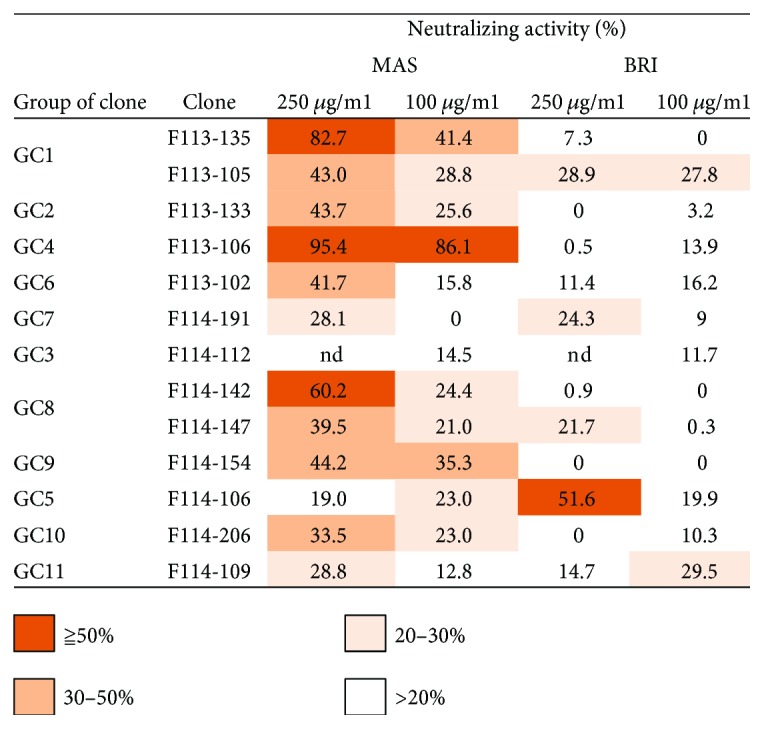
Neutralizing activity. The virus-neutralizing activity of 100 or 250 *μ*g/ml Fab-pp Ab against B/Massachusetts/2/2012 (MAS) and B/Brisbane/60/2008 (BRI) viruses was examined by the focus reduction test. The focus reduction rate is shown as a percentage. nd indicates not done.

The neutralizing activity of 11 clones was examined by a focus reduction assay. In this experiment, monovalent Fab form was used. We had obtained the following results in the previous study as follows [8]. The concentration of IgG form of Ab required for 50% reduction in focus formation was about 0.01–0.03 nM. On the other hand, monovalent Fab form of Ab gave a thousand to several thousand-fold lower neutralizing activity. Therefore, we considered that it showed neutralizing activity when higher than 30% reduction at 100 *μ*g/ml (it corresponds to 1.5 *μ*M) was observed. According to this criterion, four clones that possessed HI activity, F113-135, F113-105, F113-133, and F113-106, showed neutralizing activity (Figure 10). Type 2 Abs, three clones, F114-142, F114-154, and F114-147, showed neutralizing activity. In the case of type 3 Abs, we could not obtain any conclusion. The role of type 3 Abs in protection against influenza viruses should be clarified in future experiments.

## 4. Discussion

**Figure 11:**
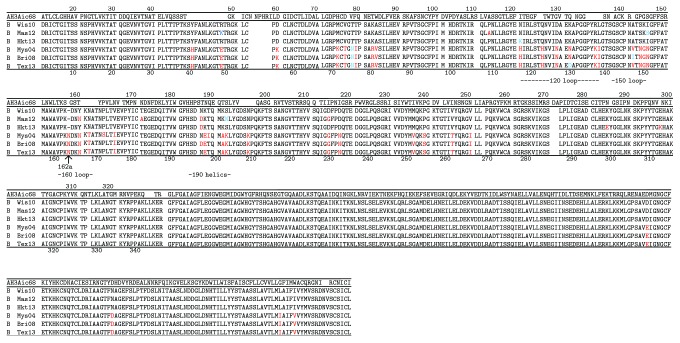
Comparison of amino acid sequences of HAs among three Yamagata lineage strains and three Victoria lineage strains of influenza B viruses. The HA sequence of A/Aichi/2/68 (H3N2 strains of influenza A virus) indicated on the top as a standard comparison.

In the present study, we analyzed the Abs against influenza B viruses induced by vaccination with seasonal viruses in one person.  Figure 11 indicates the amino acid sequences of HAs of three Yamagata lineage viruses and three Victoria lineage viruses used in the present study. The HA sequence of A/Aichi/2/68 is indicated on the top as a standard for comparison [18]. The number of respective amino acid residue for the H3 molecule is indicated at the top line. The numbers of HAs for B virus are indicated at the bottom. The difference in length between Yamagata lineage viruses and Victoria lineage viruses occurs at one locus. Between residues 162 and 163, insertion of one amino acid is observed in Victoria lineage viruses. When the amino acid sequences were compared between Yamagata lineage viruses and Victoria lineage viruses, large differences were found in five regions, 71–81, 116–137, 146–150, 162a–174, and 196–208. Except for 71–81, they are located in the surrounding regions of a sialic acid-binding pocket as follows: 120 loop (116–137), 150 loop (141–150), 160 loop (162–167), and 190 helix (194–202). For comparison, we used two mouse anti-HA mAbs whose epitopes had been determined by isolation of escape mutants. In the case of mAb 3A12, three residues, 141, 147, and 148, form a part of the epitope [10]. They are located at 150 loop. In the case of mAb 10B8, three residues, 164, 165, and 203, form a part of the epitope [12]. They are located at 160 loop. Since GC1 and GC2 Abs classified into type 1 competed well with 3A12 in the competitive ELISA, 150 loop should be a part of the epitope. While three loops and one helix regions located in the surrounding region of the receptor-binding pocket are highly immunogenic, 63% of isolated clones in the present study belonged to GC1 (Table 1).

The amino acid residues that differ between Yamagata lineage and Victoria lineage viruses are mainly located in the restricted regions that correspond to the highly immunogenic regions. On the other hand, the degree of differences in these regions among the viruses classified into the respective lineages is low. Therefore, in human immune systems, it will be relatively easy to generate cross-reactive Abs among the respective lineage viruses, but it should be difficult to generate cross-reactive Abs that react to both lineage viruses. In the case of F113-106, which was classified into type 2 but showed HI activity, it competed slightly with 3A12 in the competitive ELISA. Although different amino acids are observed in many residues located in all three loops and one helix, isolation of F113-106 suggested that there is a common epitope among influenza B viruses even in a surrounding region of a receptor-binding pocket. In fact, CR8033 isolated by Dreyfus et al. [19] neutralized both Yamagata and Victoria lineage viruses and showed HI activity against Yamagata lineage viruses. While F113-102 also showed HI activity, the concentration of Ab required for the inhibition was very high.

CR8071, isolated by Dreyfus et al. [19], also neutralized both Yamagata lineage and Victoria lineage viruses but did not show HI activity. We isolated nine clones belonging to GC3 to 9, except for GC4, classified into type 2 which showed characteristics similar to that of CR8071 and reported the evidence showing there are at least four epitopes that commonly present on HAs of both Yamagata and Victoria lineage viruses. While the epitope of CR8071 was shown by X-ray analysis, we noticed the following. CR8071 used V_H_1-18 gene and V*_λ_*1-47 genes as germline genes for the H chain and L chain, respectively. Since F114-142 and F114-147 also utilized these two germline genes (Figures 1 and 2), it may be possible that the epitope recognized by F114-142 and F114-147 is the same as that by CR8071.

CR9114, isolated by Dreyfus et al. [19], is the only Ab that can bind to the HA of both influenza A and B viruses among the Abs isolated to date. The epitope on HA is composed of three portions, 38–42 in the N terminal portion of HA1, 291–293 in the C terminal portion of HA1, and moreover, 18–21, 38–46, and 49–53 in the HA2. CR9114 used V_H_1-69 gene, and the Ag-binding site is composed of HCDR1, HCDR2, and HCDR3, and FR3 of H chain without contribution of the V_L_ domain. On the other hand, in the case of F114-206 and F114-109 that bind to HA of both A and B viruses, they utilized V_H_3-7 gene for the H chain and V*_λ_*1-44 or V*_λ_*1-47 gene for the L chain (Figures 1 and 2). Therefore, it is likely that both H and L chains are involved in forming the Ag-binding site. Thus, the epitope recognized by these Abs may be different from that recognized by CR9114.

Based on the antigenic properties of HA, influenza A viruses are categorized into two groups: group1 composed of 12 subtypes (H1, H2, H5, H6, H8, H9, H11, H12, H13, H16, H17, and H18) and group 2 composed of 6 subtypes (H3, H4, H7, H10, H14, and H15) [20]. Since Abs encoded by the V_H_1-69 gene that can neutralize all group 1 viruses were discovered [21, 22], many broadly neutralizing Abs (bnAbs) have been isolated at various levels, including group 2 viruses [23] and influenza A [24] and B viruses [18, 25]. In the present study, we isolated Abs that might bind to HAs of both A and B influenza viruses. While the role of these broadly reacting Abs in protection against influenza viruses has not been clarified yet, the following factors should be crucial in the synthesis of memory B cells that produce bnAbs. The most fundamental factor is the presence of common epitope(s) among viruses belonging to a certain type, group, or lineage. The second factor is the immunogenicity of such epitopes in the human immune system. This study indicated the presence of common epitope(s) at three different levels. It has been shown that the common epitope present among Yamagata lineage viruses is immunogenic, and Abs binding to this epitope prevent interaction between HA and sialic acid [11, 18]. We showed the presence of at least four common epitopes on HAs of both Yamagata and Victoria lineage viruses recognized by type 2 Abs without HI activity. Most of these Abs showed neutralizing activity in the focus reduction assay. The isolation of Abs belonging to GC10 to 13 classified into type 3 suggested the presence of common epitope(s) on HAs of influenza A and B viruses. Since we analyzed the Ab repertoire of only one person, it is still possible that the presence of Abs that bind to the common epitope on HA of both influenza A and B viruses is observed only in specific persons and may not be a general phenomenon.

## 5. Conclusions

Broadly reacting anti-HA Abs against influenza B viruses of Yamagata lineage were isolated at three different levels, Yamagata lineage viruses, both Yamagata and Victoria lineage viruses, and both influenza A and B viruses. These results indicated that common epitopes are present at these three levels and they could be immunogenic in humans.

## Figures and Tables

**Table 1:** Classification of isolated clones.

Antigen	Group of clone	Representative clone	Number of isolated clone	Total
MAS^a^	GC1	F113-135	16	81
		F113-140	4	
		F113-103	12	
		F113-162	3	
		F113-104	4	
		F113-105	13	
		F113-110	4	
		F113-139	4	
		F113-126	9	
	GC2	F113-133	1	
	GC3	F113-182	1	
	GC4	F113-101	5	
		F113-106	3	
	GC5	F113-146	1	
	GC6	F113-102	1	

BRI^b^	GC7	F114-191	1	29
	GC8	F114-142	1	
		F114-147	1	
	GC3	F114-112	3	
	GC9	F114-154	1	
	GC5	F114-106	10	
	GC10	F114-206	1	
	GC11	F114-109	2	
	GC12	F114-123	6	
		F114-195	2	
	GC13	F114-197	1	

^a^B/Massachusetts/2/2012. ^b^B/Brisbane/60/2008.

**Table 2:** HI activity.

Group of clone	Clone	HI titer (*μ*g/ml)	
		MAS^a^	BRI^b^
GC1	F113-135	5	—
	F113-103	5	—
	F113-104	5	—
	F113-105	5	—
	F113-110	5	—
	F113-139	5	—
	F113-126	5	—
GC2	F113-133	5	—
GC3	F113-182	>200	—
GC4	F113-101	5	—
	F113-106	5	—
GC6	F113-102	50	—
GC7	F114-191	—	>200
GC8	F114-142	—	>200
GC3	F114-112	—	>200
GC9	F114-154	—	>200
GC5	F114-106	—	>200
GC10	F114-206	—	>200
GC11	F114-109	—	>200
GC12	F114-123	—	>200
	F114-195	—	>200
GC13	F114-197	—	>200

^a^B/Massachusetts/2/2012. ^b^B/Brisbane/60/2008.

## Abbreviations

Ab: Antibody
Ag: Antigen
bnAbs: Broadly neutralizing antibodies
BSA: Bovine serum albumin
CDR: Complementarity-determining region
ELISA: Enzyme-linked immunosorbent assay
FFU: Focus-forming unit
H: Heavy
HA: Hemagglutinin
HI: Hemagglutination inhibition
HRP: Horse radish peroxidase
L: Light
mAb: Monoclonal antibody
MEM: Minimum essential media
PBS: Phosphate-buffered saline
PCR: Polymerase chain reaction
PFA: Paraformaldehyde
PVDF: Polyvinylidene difluoride
SDS-PAGE: Sodium dodecyl sulfate-polyacrylamide gel electrophoresis
TMB: 3,3′,5,5′-Tetramethylbenzidine.
